# Longitudinal Analysis of CCR5 and CXCR4 Usage in a Cohort of Antiretroviral Therapy-Naïve Subjects with Progressive HIV-1 Subtype C Infection

**DOI:** 10.1371/journal.pone.0065950

**Published:** 2013-06-18

**Authors:** Martin R. Jakobsen, Kieran Cashin, Michael Roche, Jasminka Sterjovski, Anne Ellett, Katharina Borm, Jacqueline Flynn, Christian Erikstrup, Maelenn Gouillou, Lachlan R. Gray, Nitin K. Saksena, Bin Wang, Damian F. J. Purcell, Per Kallestrup, Rutendo Zinyama-Gutsire, Exnevia Gomo, Henrik Ullum, Lars Østergaard, Benhur Lee, Paul A. Ramsland, Melissa J. Churchill, Paul R. Gorry

**Affiliations:** 1 Centre for Virology, Burnet Institute, Melbourne, Victoria, Australia; 2 Centre for Immunology, Burnet Institute, Melbourne, Victoria, Australia; 3 Centre for Population Health, Burnet Institute, Melbourne, Victoria, Australia; 4 Department of Infectious Diseases, Aarhus University, Aarhus, Denmark; 5 Department of Clinical Immunology, Aarhus University, Aarhus, Denmark; 6 Department of Surgery (Austin Health), University of Melbourne, Victoria, Australia; 7 Department of Microbiology and Immunology, University of Melbourne, Victoria, Australia; 8 Department of Biochemistry and Molecular Biology, Monash University, Melbourne, Victoria, Australia; 9 Department of Medicine, Monash University, Melbourne, Victoria, Australia; 10 Department of Immunology, Monash University, Melbourne, Victoria, Australia; 11 Department of Microbiology, Monash University, Melbourne, Victoria, Australia; 12 Department of Infectious Diseases, Monash University, Melbourne, Victoria, Australia; 13 Department of Microbiology, Immunology, and Molecular Genetics, David Geffen School of Medicine, University of California Los Angeles, Los Angeles, California, United States of America; 14 Center for Virus Research, Westmead Millennium Institute, Westmead, New South Wales, Australia; 15 Department of Clinical Immunology, Rigshospitalet, Copenhagen, Denmark; 16 National Institute of Health Research, Harare, Zimbabwe; The University of Hong Kong, Hong Kong

## Abstract

HIV-1 subtype C (C-HIV) is responsible for most HIV-1 cases worldwide. Although the pathogenesis of C-HIV is thought to predominantly involve CCR5-restricted (R5) strains, we do not have a firm understanding of how frequently CXCR4-using (X4 and R5X4) variants emerge in subjects with progressive C-HIV infection. Nor do we completely understand the molecular determinants of coreceptor switching by C-HIV variants. Here, we characterized a panel of HIV-1 envelope glycoproteins (Envs) (n = 300) cloned sequentially from plasma of 21 antiretroviral therapy (ART)-naïve subjects who experienced progression from chronic to advanced stages of C-HIV infection, and show that CXCR4-using C-HIV variants emerged in only one individual. Mutagenesis studies and structural models suggest that the evolution of R5 to X4 variants in this subject principally involved acquisition of an “Ile-Gly” insertion in the gp120 V3 loop and replacement of the V3 “Gly-Pro-Gly” crown with a “Gly-Arg-Gly” motif, but that the accumulation of additional gp120 “scaffold” mutations was required for these V3 loop changes to confer functional effects. In this context, either of the V3 loop changes could confer possible transitional R5X4 phenotypes, but when present together they completely abolished CCR5 usage and conferred the X4 phenotype. Our results show that the emergence of CXCR4-using strains is rare in this cohort of untreated individuals with advanced C-HIV infection. In the subject where X4 variants did emerge, alterations in the gp120 V3 loop were necessary but not sufficient to confer CXCR4 usage.

## Introduction

More than 33 million people are infected with human immunodeficiency virus (HIV) and around 20 million have died from AIDS. Approximately 2.1 million new infections occur annually [1] and most of these individuals live in developing countries with limited access to potentially life saving antiretroviral therapies. Moreover, HIV is predicted to become the leading burden of disease in middle and low-income countries by 2015 [2].

Genetically, HIV type 1 (HIV-1) consists of groups M (Main), N (New) and O (Outlier) [3], with group M viruses accounting for >32 million HIV-1 cases. The spread of HIV-1 in humans has enabled the evolution of group M viruses into a number of distinct subtypes (A-D, F-H, J, K) and intersubtype recombinant forms. Subtype C HIV-1 (C-HIV) is spreading rapidly and now accounts for >50% of infections worldwide and >95% of infections in southern Africa and central Asia (reviewed in [4]), which are regions of the world burdened with the overwhelming majority of HIV-1 infections.

Several aspects of HIV-1 pathogenesis are influenced by the mechanism of HIV-1 entry into target cells, including viral tropism, HIV-1 transmission and progression, and responsiveness to HIV-1 entry inhibitors (reviewed in [5], [6]). HIV-1 entry is mediated by the viral envelope glycoproteins (Env), which comprise surface gp120 glycoproteins non-covalently linked to transmembrane gp41 glycoproteins that embed the complex into the viral membrane [7], [8], [9], and is initiated by the interaction between gp120 and cellular CD4. This interaction occurs with high affinity [10], and induces conformational changes in gp120 resulting in exposure of the binding site for a cellular coreceptor, either CCR5 or CXCR4 (reviewed in [11], [12]). Coreceptor binding by the gp120-CD4 complex triggers further conformational changes in Env, leading to a structural rearrangement in gp41 that enables fusion between the viral and cellular membranes, and entry of the virion core into the host cell.

Although C-HIV is spreading rapidly, paradoxically C-HIV is less virulent than other HIV-1 subtypes *ex vivo* [3], [13] suggesting unique molecular mechanisms that simultaneously impair fitness and facilitate favorable transmission events. However, relatively little is known about the pathogenesis of C-HIV. During subtype B HIV-1 (B-HIV) infection, viruses that use CCR5 as the coreceptor for HIV-1 entry (R5 strains) predominate at early stages of infection, but viral variants that have acquired the ability to use CXCR4 instead of CCR5 (X4 strains) or together with CCR5 (R5X4 strains) emerge in 40 to 50% of subjects and accelerate the rate of disease progression [14], [15]. This is, in part, due to the expanded repertoire of CXCR4-expressing T-cells available for infection [16]. In contrast, the available data suggest that C-HIV pathogenesis is driven principally by R5 HIV-1 viruses, with X4 and R5X4 variants detected infrequently (reviewed in [3], [4]). However, these conclusions have been based principally on cross-sectional studies of chronically-infected subjects, studies of early/acute infected individuals, relatively small studies of late stage C-HIV infection where subjects were ART-experienced which likely altered the natural history of the disease, or studies which relied on primary C-HIV isolates where passage in PBMC may have resulted in a selection bias [17], [18], [19], [20], [21], [22], [23], [24], [25], [26], [27], [28], [29]. The extent to which X4 and/or R5X4 C-HIV variants emerge at later stages of infection and influence the natural history of C-HIV pathogenesis is therefore yet to be firmly established, and the precise molecular mechanisms underlying the emergence of X4 and R5X4 C-HIV strains are unknown.

Detailed, longitudinal studies of C-HIV evolution from chronic to advanced stages of infection in clinically well-characterized subjects are lacking, but are essential for understanding the role of coreceptor specificity alterations in C-HIV pathogenesis. Here, we generated and characterized a large panel of functional HIV-1 Envs (n = 300) cloned directly from longitudinally-collected plasma samples of 21 antiretroviral therapy (ART)-naïve subjects from rural Zimbabwe, who experienced progression from chronic to advanced stages of C-HIV infection over an approximately 3 year period. In these subjects, the development of phenotypically-verified CXCR4-using variants that were capable of entering primary CD4+ T-cells via CXCR4 was exceedingly rare, with such variants detected at advanced infection in only one subject. In contrast, R5 C-HIV strains were maintained almost exclusively from chronic to advanced infection in 20/21 subjects. Finally, with Env mutagenesis and structural modeling of gp120 we show that the determinants of coreceptor switching from R5 to X4 variants principally involved the accumulation of two distinct mutations in the gp120 V3 loop, most likely conferring their functional effects in the context of additional gp120 “scaffold” mutations. Together, these findings provide new insights into the natural history of progressive C-HIV infection, which will be important to consider in the development of targeted approaches to treat and prevent C-HIV infection.

## Materials and Methods

### Ethics

Written informed consent was provided by the subjects for the use of stored plasma samples. Ethics approval for the use of these samples was granted by the Medical Research Council of Zimbabwe (MRCZ/A/918) and by the Central Medical Scientific Ethics Committee of Denmark (624-01-0031).

### Cells

JC53 [30], U87-CD4 [31], U87-CD4/CCR5 [14], U87-CD4/CXCR4 [14], NP2-CD4 [32], NP2-CD4/CCR5 [33], NP2-CD4/CXCR4 [33] were maintained as described in the indicated references. Peripheral blood mononuclear cells (PBMC) were purified from healthy donors, stimulated and activated with PHA/IL-2 and cultured as described previously [34].

### PCR Amplification, HIV-1 Env Cloning, and Identification of Functional Envs

Viral RNA was purified from plasma using a QIAamp Viral RNA Mini kit (Qiagen) according to the manufacturers’ protocol. The full-length HIV-1 *env* gene was amplified in a one-step reverse transcription (RT)-PCR reaction using SuperScript III reverse transcriptase (Invitrogen) and Platinum *Taq* high-fidelity DNA polymerase and primers Env_fwd_ (5′-GAGCAGAAGACAGTGGCAATGAGAGTGA-3′) and Env/Nef_rev_ (5′-GGCGTTCCAGGAGGAGGGGAC-3′). The RT-PCR cycling consisted of an initial incubation at 45°C for 45 min then a denaturation step at 94°C for 2 min, followed by 35 cycles of 94°C for 15 s, 56°C for 30 s and 68°C for 2 min, then a final extension at 68°C for 5 min. The second round amplification with primers Env-*Kpn*I and Env-*Bam*HI [35], subsequent cloning into the pSVIII-Env expression plasmid [36], and identification of functional Envs using Env-pseudotyped GFP-reporter viruses was carried out as described previously [34], [37], [38], [39].

### Production and Titration of Env-pseudotyped Luciferase Reporter Viruses

Env-pseudotyped, luciferase reporter viruses were produced by transfection of 293T cells with pCMVΔP1ΔenvpA, pHIV-1Luc and pSVIII-Env plasmids at a ratio of 1∶3:1 using Lipofectamine 2000 (Invitrogen), as described previously [37], [38], [40], [41], [42], [43], [44]. Supernatants were harvested 48 h later, clarified by filtration through 0.45 µM filters, aliquotted and stored at −80°C. The TCID_50_ of virus stocks was determined by titration in JC53 cells [30], as described previously [34], [38], [42], [44].

### HIV-1 Entry Assays

The ability of Env-pseudotyped luciferase reporter viruses to use CCR5 and/or CXCR4 was determined by single-round entry assays using two independent cell systems (U87 [31] and NP2 [33]), which stably express CD4 together with CCR5 or CXCR4, as described previously [41]. Briefly, 1×10^4^ cells were inoculated with 5-fold serial dilutions of virus for 6 h at 37°C. Cells were then media changed and incubated a further 48 h at 37°C. HIV-1 entry was then measured by assaying luciferase activity in cell lysates (Promega), according to the manufacturers’ protocol. The negative controls used to determine the background level of luciferase activity included mock-infected cells treated with culture medium instead of virus, and cells inoculated with luciferase reporter virus pseudotyped with the non-functional ΔKS Env [45]. The level of virus entry was scored as – (<5 fold above background), + (5–50 fold above background), ++ (50–300 fold above background), or +++ (>300 fold above background). Any detection of CXCR4 usage by the C-HIV Envs was confirmed by repeated assays in the presence of the CXCR4 inhibitor AMD3100. The measurement of HIV-1 entry and coreceptor preference in PBMC was conducted as described previously [34], [42].

### Env Sequencing and Phylogenetic Analysis

Envs were sequenced by Big Dye terminator sequencing and analyzed using a model 3130 Genetic Analyzer (Applied Biosystems). Env nucleotide sequences (within amino acid positions 6348 and 8478 relative to the HXB2 strain of HIV-1) were aligned against the corresponding regions of a panel of reference sequences from different HIV-1 subtypes (obtained from the Los Alamos HIV Database) using ClustalW. Phylogenetic analysis was conducted by the Neighbor-joining method using MEGA4 software [46], with bootstrap resampling done with 1000 replicates. Evolutionary distances were computed using the maximum composite-likelihood method with complete deletion option, that has been optimised for more accurately inferring large phylogenies [47].

### Structural Modeling of gp120

Three-dimensional protein structures of representative “enrolment” (1109-E-10) and “final” (1109-F-30) gp120 sequences derived from subject 1109, and those of various 1109-F-30 V3 loop mutants, were prepared using the Discovery Studio suite, version 3.0 (Accelrys, San Diego, CA) as described previously [41], [42], [44], [48], [49], using the crystal structure of CD4-bound gp120 containing the V3 variable loop docked with the nuclear magnetic resonance (NMR) structure of a sulfated N-terminal peptide of CCR5 (residues 2 to 15) (kindly provided by P. D. Kwong [50]) as a template. Homology models of gp120 bound to a sulfated CXCR4 N-terminal peptide were generated as described previously [42]. Briefly, the CCR5 peptide sequence (SPIY^10^DINYY^15^) was mutated to the CXCR4 N-terminus sequence (ISIY^7^TSDNY^12^) using a sequence alignment, with conserved sulfated tyrosine residues numbered. Harmonic restraints were applied prior to optimization using the Steepest Descent energy minimization protocol, which incorporates iterative cycles of conjugate-gradient energy minimisation against a probability density function that includes spatial restraints derived from the template and residue specific properties [51].

### Env Mutagenesis

All gp120 mutants were synthesized by GenScript Pty. Ltd. (Piscataway, NJ, USA), and subcloned into the pSVIII-Env expression vector [36]. The authenticity of the gp120 mutants was verified by full-length sequencing. The Env mutants consist of the 1109-F-30 Env with Pro318 of the 1109-E-10 Env (M1), 1109-F-30 with deletion of the Ile314-Gly315 insertion (M2), 1109-F-30 with Pro318 of the 1109-E-10 Env and deletion of the Ile314-Gly315 insertion (M3), 1109-F-30 with Thr329 of 1109-E-10 (M4), 1109-F-30 with Asn331 of 1109-E-10 (M5), 1109-F-30 with the whole V1 loop of 1109-E-10 (M6), 1109-F-30 with the V1 loop and Pro318 of 1109-E-10 (M7), 1109-F-30 with the V1 loop of 1109-E-10 and deletion of the Ile314-Gly315 insertion (M8), 1109-F-30 with the V1 loop and Thr329 of 1109-E10 (M9), 1109-F-30 with the V1 loop and Asn331 of 1109-E-10 (M10), 1109-F-30 with the V1 loop and Pro318 of 1109-E-10 and deletion of the Ile314-Gly315 insertion (M11), 1109-F-30 with the V1 loop, Pro318 and Thr329 of 1109-E-10 (M12), and 1109-F-30 with the V1 loop, Pro318 and Asn331 of 1109-E-10 (M13). In addition, we produced an Env mutant of 1109-F-30 containing the whole V3 loop of 1109-E-10 (M14), and a mutant of 1109-F-30 containing both the V3 and V1 loops of 1109-E-10 (M15). Additional Env mutants produced include 1109-E-10 with the Ile314-Gly315 insertion present in 1109-F-30 (M16), 1109-E-10 with Arg318 of the 1109-F-30 Env (M17), and 1109-E-10 with both the Ile314-Gly315 insertion and Arg318 of the 1109-F-30 Env (M18).

### Nucleotide Accession Numbers

Env nucleotide sequences have been assigned GenBank accession numbers HQ707833 to HQ708154 (see also Table S1).

## Results

### Establishment of a Longitudinal Cohort of Subjects Experiencing Progressive C-HIV Infection

**Figure 1:**
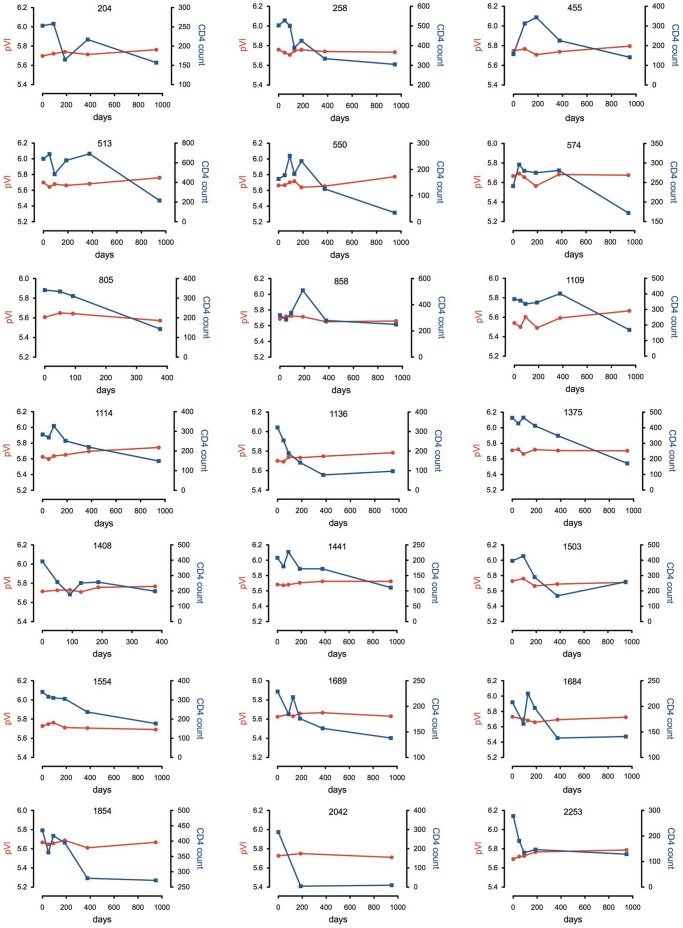
Changes in plasma viral load and CD4+ T-cell count over time in each of the study subjects. Plasma viral load (pVl) is shown as Log10 HIV-1 RNA copies/ml. CD4+ T-cell count is shown as cells/µL.

Twenty-one subjects were selected from the clinically well-characterized Mupfure schistosomiasis and HIV (MUSH) cohort from rural Zimbabwe [52], [53], who showed clinical and immunological evidence of progression from chronic to advanced stages of HIV-1 infection over an approximately 3-year period between 2001 and 2004. The Karnofsky scale of performance (KPS) score and CDC status of the subjects at study enrolment, and plasma viral load and CD4+ T-cell counts over time are shown in Table 1. The selected subjects showed notable declines in CD4+ T-cell count. The changes in plasma viral load and CD4+ T-cell count over time for each subject are shown in more detail in Figure 1. All but three subjects (204, 455, 1503) had schistosomiasis, which was treated with a single oral dose of praziqantel at study enrolment [54]. Because the national ART program in Zimbabwe was not effectively implemented until 2005, all subjects were ART-naïve throughout the study period. This enabled a rare opportunity to characterize adaptive alterations that occur during progressive C-HIV infection without the influence of antiretroviral intervention.

**Table 1:** Clinical characteristics of the study subjects and laboratory measurements.

Subject ID	Sex	Age	KPS Score	CDC Status	Plasma viral load (RNA copies/ml)				CD4+ T-cell count (cells/µl)			ΔViral load	ΔCD4+ T-cell count
					T_Enrol_	T_Inter_	T_Final_		T_Enrol_	T_Inter_	T_Final_		
204	F	26	100	A	5.70	5.75	5.76		253	217	157	0.064	−96
258	F	30	100	A	5.76	5.74	5.73		503	334	305	0.025	−198
455	F	44	70	A	5.75	5.74	5.80		157	313	141	0.048	−172
513	F	46	100	B	5.70	5.66	5.76		640	624	216	0.06	−424
550	F	31	100	B	5.66	5.65	5.77		164	126	35	0.11	−129
574	F	27	100	A	5.67	5.68	5.68		241	281	172	0.009	−69
805	F	40	100	A	5.61	5.57	N/A		341	143	N/A	−0.034	−198
858	F	27	90	B	5.69	5.65	5.66		320	280	250	−0.027	−70
1109	F	26	100	A	5.54	5.59	5.67		367	402	169	0.125	−198
1114	F	35	100	B	5.63	5.70	5.74		284	220	149	0.118	−135
1136	F	29	100	A	5.70	5.75	5.78		231	78	97	0.08	−134
1375	F	22	100	B	5.71	5.71	5.70		464	349	171	−0.006	−293
1408	F	27	100	A	5.72	5.77	N/A		392	198	N/A	0.05	−197
1441	M	28	100	B	5.68	5.73	5.73		208	170	111	0.04	−97
1503	F	29	100	A	5.73	5.69	5.71		396	168	259	−0.016	−137
1554	F	28	50	C	5.73	5.71	5.69		342	237	177	−0.036	−165
1684	F	40	100	A	5.73	5.69	5.72		208	138	141	−0.006	−67
1689	M	34	100	A	5.63	5.67	5.63		229	157	138	0.005	−91
1854	F	31	100	A	5.61	5.67	5.67		435	279	272	0.001	−163
2042	F	41	100	A	5.73	5.75	5.71		278	5	10	−0.015	−277
2253	F	42	100	B	5.69	5.77	5.79		278	147	129	0.09	−149

Age, Karnofsky scale of performance (KPS) score and Centers for Disease Control (CDC) status were determined at study enrolment. Plasma viral load values are shown as log10. Plasma viral load and CD4+ T-cell counts are shown at the times of study enrolment (T_enrol_), approximately 1 year later (T_inter_), and approximately 3 years after enrolment (T_final_). The change in viral load (Δviral load) and CD4+ T-cell count (Δ CD4+ T-cell count) over the study period is also shown. F, female; M, male; N/A, not available.

### Cloning and Characterization of Functional C-HIV Envs

**Figure 2:**
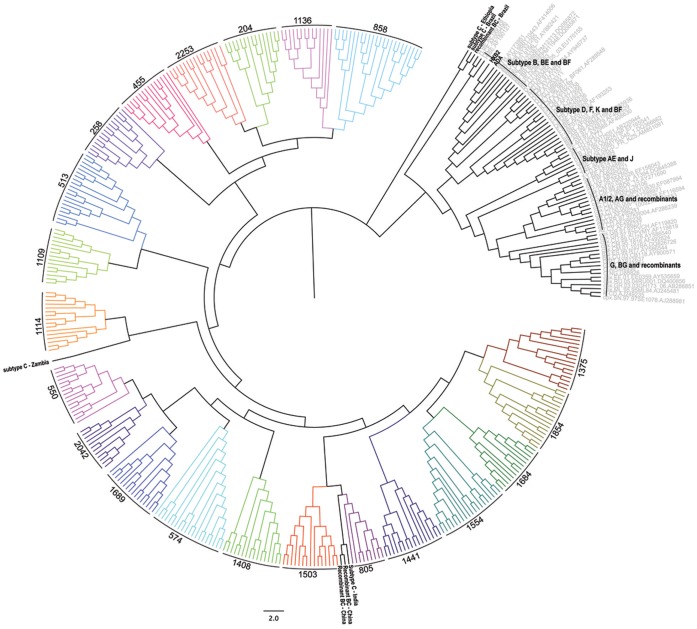
Inter-subject phylogenetic relationships of cloned Env sequences. Phylogenetic analysis was conducted on the entire Env sequence set as described in the Materials and Methods, confirming that the sequences cloned from the patient plasma samples are HIV-1 subtype C. The data also demonstrate no inter-sample mixing of Env sequences.

Stored plasma samples that were collected at study enrolment (T_enrol_), approximately 1 year later (T_inter_), and approximately 3 years after enrolment (T_final_) (Table 1) were used to amplify and clone the gp160 coding region of HIV-1 Env into the pSVIII-Env expression plasmid. Between 2 and 8 functional Envs from each plasma sample, totalling 300 Envs across the cohort, were identified based on the ability to support the entry of Env-pseudotyped GFP reporter viruses into CD4/CCR5/CXCR4-expressing JC53 cells (data not shown). Envs were sequenced and aligned against a panel of reference sequences from different HIV-1 subtypes and subjected to phylogenetic analysis, which demonstrated clustering among reference C-HIV sequences and separation from sequences of other HIV-1 subtypes (Figure 2). The Env sequences from each subject congregated in distinct monophyletic clusters with no inter-subject mixing. The intra-subject phylogenetic relationships of Env sequences are shown in greater detail in Figure S1. Therefore, we established an extensive, longitudinal bank of functional Envs derived from circulating viral strains of ART-naïve subjects progressing from chronic to advanced stages of C-HIV infection.

### The Emergence of CXCR4-using C-HIV Strains is Rare during Progressive, Untreated Infection

To determine coreceptor usage, we produced luciferase reporter viruses pseudotyped with each of the 300 Envs and conducted single-round entry assays in cell lines expressing CD4 and CCR5 or CXCR4. Two independent cell systems were used in these experiments (U87 and NP-2) [31], [33]. Positive controls included luciferase reporter viruses pseudotyped with well characterized reference B-HIV R5 (YU2, JRCSF, ADA, NB6-6, NB8-57), X4 (HXB2) and R5X4 (89.6, C2-22, Macs1-Spln-12) Envs which, as expected from the results of previous studies [34], [37], [38], [55], [56], [57], used CCR5-only, CXCR4-only, or both coreceptors for entry, respectively (Table S1; parts 6 and 7). The efficiency of the individual C-HIV Envs to use CCR5 and/or CXCR4 for HIV-1 entry compared to reference B-HIV Envs, and their corresponding gp120 V3 sequences, is shown in Table S1 (parts 1 to 5), and a summary of the coreceptor usage results is presented in Table 2. Our results show that 19/21 subjects (∼90%) harbored C-HIV to advanced stages of infection where only R5 Envs were detectable. R5X4 Envs were detected as minor variants in one subject (1854), and X4 Envs emerged as the dominant variant at late stage infection in another subject (1109). Although the CXCR4-usage of the R5X4 Envs from subject 1854 could be completely inhibited by the CXCR4 antagonist AMD3100 in U87-CD4/CXCR4 and NP2-CD4/CXCR4 cells (Table S1), the ability of these Envs to use CXCR4 was very weak compared to their usage of CCR5 (Table S1), and studies of coreceptor preference in primary cells showed that they use CCR5 exclusively for HIV-1 entry into PBMC (data not shown). Furthermore, these Envs are scored as R5-like by bioinformatic coreceptor usage prediction programs (Geno-2-Pheno, subtype C Web PSSM, and CoRSeq_V3-C_ [58]). Consistent with the latter observation, the intra subject Env sequence alterations segregating these R5X4 Envs from R5 Envs occur in gp41, rather than the V3 loop region of gp120 (data not shown). In contrast, the X4 Envs from subject 1109 had very efficient CXCR4 usage in the indicator cell lines compared to reference B-HIV X4 and R5X4 Envs (Table S1), used CXCR4 for entry into PBMC (data not shown), and are scored as X4-like by the abovementioned coreceptor usage prediction programs. Thus, we consider that the minor R5X4 variants that were detected in subject 1854 are ostensibly CCR5-restricted, and that functionally relevant CXCR4-using Envs only emerged in one subject (1109). Together, our results, which reflect the natural history of C-HIV from chronic to advanced stages of infection, suggest that C-HIV pathogenesis is indeed driven principally by R5 viral strains, with functionally-relevant CXCR4-using variants detected very infrequently in our cohort.

**Table 2:** Summary of coreceptor usage.

Subject ID	Enrolment (T_Enrol_)	Intermediate (T_Inter_)	Final (T_Final_)	Summary of phenotypes
204	R5(n = 5 clones)	R5(n = 3 clones)	R5(n = 5 clones)	R5
258	R5(n = 5 clones)	R5(n = 5 clones)	R5(n = 6 clones)	R5
455	R5(n = 4 clones)	R5(n = 6 clones)	R5(n = 6 clones)	R5
513	R5(n = 2 clones)	R5(n = 5 clones)	R5(n = 7 clones)	R5
550	R5(n = 4 clones)	R5(n = 5 clones)	R5(n = 5 clones)	R5
574	R5(n = 3 clones)	R5(n = 5 clones)	R5(n = 8 clones)	R5
805	R5(n = 3 clones)	N/A	R5(n = 4 clones)	R5
858	R5(n = 7 clones)	R5(n = 6 clones)	R5(n = 7 clones)	R5
1109	R5(n = 5 clones)	R5(n = 5 clones)	X4(n = 6 clones)	R5/X4
1114	R5(n = 5 clones)	R5(n = 5 clones)	R5(n = 6 clones)	R5
1136	R5(n = 4 clones)	R5(n = 5 clones)	R5(n = 5 clones)	R5
1375	R5(n = 6 clones)	R5(n = 6 clones)	R5(n = 3 clones)	R5
1408	R5(n = 6 clones)	N/A	R5(n = 8 clones)	R5
1441	R5(n = 6 clones)	R5(n = 4 clones)	R5(n = 4 clones)	R5
1503	R5(n = 3 clones)	R5(n = 5 clones)	R5(n = 7 clones)	R5
1554	R5(n = 4 clones)	R5(n = 4 clones)	R5(n = 7 clones)	R5
1684	R5(n = 4 clones)	R5(n = 4 clones)	R5(n = 4 clones)	R5
1689	R5(n = 4 clones)	R5(n = 6 clones)	R5(n = 4 clones)	R5
1854	R5(n = 3 clones)	R5/R5X4(n = 4/1 clones)	R5/R5X4(n = 6/1 clones)	R5/R5X4
2042	R5(n = 3 clones)	R5(n = 4 clones)	R5(n = 4 clones)	R5
2253	R5(n = 4 clones)	R5(n = 6 clones)	R5(n = 3 clones)	R5

The numbers in parentheses represent the numbers of Env clones that display R5, R5X4 or X4 phenotype as described in the Materials and Methods. Coreceptor usage results for individual Env clones are shown in more detail in . N/A, not available.

### Sequence and Structural Analysis of R5 and X4 Envs from Subject 1109

**Figure 3:**
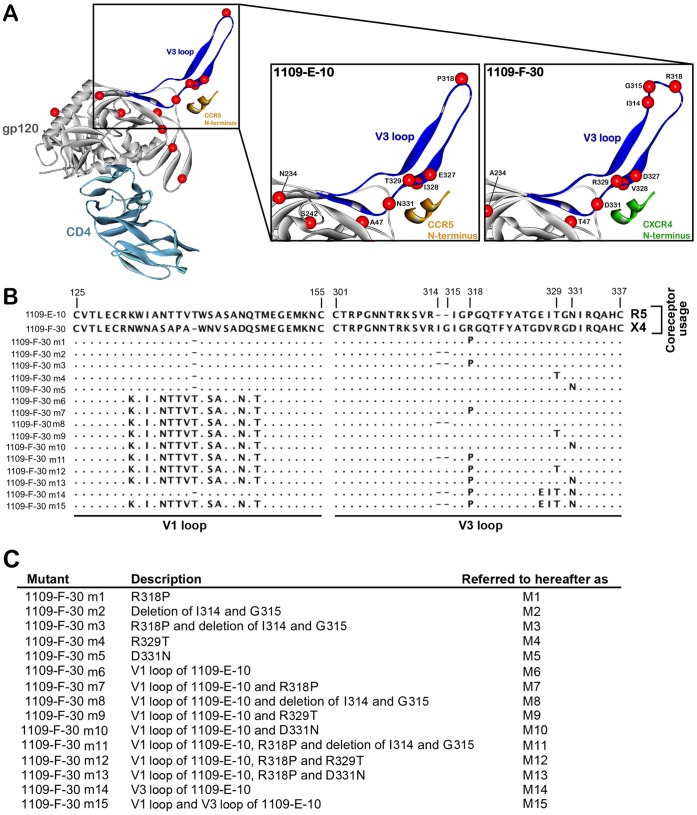
V3 loop alterations segregating X4 from R5 C-HIV Envs, and Env mutagenesis strategy. (A, left) Ribbon diagram showing a gp120 model of 1109-E-10 Env (grey) in complex with CD4 (light blue) and a sulfated CCR5 N-terminus peptide (orange). The V3 loop is highlighted in blue. The location of amino acids segregating X4 and R5 Envs from this subject shown by space-filled models of their α-carbon atoms (red spheres). (A, center) Close up view of the V3 loop of the R5 1109-E-10 gp120 bound to the CCR5 N-terminus peptide (orange), showing the R5-associated amino acids as red spheres. (A, right) Close up view of the V3 loop of the X4 1109-F-30 gp120 bound to a model of the CXCR4 N-terminus peptide (green), showing the X4-associated amino acids as red spheres. (B) Amino acid sequences of the Env mutants, aligned against the gp120 sequence of the X4 1109-F-30 sequence. Dots indicate residues identical to 1109-F-30, dashes indicate gaps. Numbers refer to amino acid positions in the V1 and V3 loop regions. (C) Brief descriptions of the Env mutants, which are provided in greater detail in Materials and Methods.

To better understand the Env sequence determinants of C-HIV coreceptor switching, we first compared the full-length gp120 sequences of the “enrolment” (R5), “intermediate” (R5), and “final” (X4) Envs from subject 1109. Notable amino acid alterations that segregated the late-emerging X4 Envs from the antecedent R5 Envs were mapped to the gp120 V1 and V3 loop regions (Fig. S2). To understand how the V3 loop alterations may potentially affect coreceptor specificity, with the view to guiding the rational design of mutagenesis experiments, we next produced homology models of representative “enrolment” (1109-E-10) and “final” (1109-F-30) gp120 proteins in their CD4-bound state interacting with peptide models of either the CCR5 or CXCR4 N-terminus, as described previously [41], [42], [44], [49]. Amino acid alterations occurring in 1109-F-30 Env clustered at the crown and stem regions of the V3 loop, and included Arg318 resulting in substitution of the highly conserved “Gly-Pro-Gly” crown motif for “Gly-Arg-Gly”, an Ile314-Gly315 insertion immediately proximal to the crown alteration, and Asp327, Val328, Arg329 and Asp331 in the descending strand of the V3 loop stem (Fig. 3A). The molecular models show that the V3 crown and stem alterations have the potential to alter the conformation of the V3 loop (Fig. 3A). Since current models of gp120 binding to coreceptor suggest that the V3 loop crown interacts with the coreceptor extracellular loop 2 region and the V3 loop stem interacts with the coreceptor N-terminus to mediate HIV-1 entry [59], [60], [61], [62], we hypothesized that combinations of these V3 crown/stem mutations contribute to the evolution of R5 to X4 variants in subject 1109, and in addition, that a subset of these alterations give rise to “transitional” R5X4 intermediates that most likely arose after the “intermediate” timepoint and disappeared before the “final” timepoint. Database analysis of published independent C-HIV Env sequences where phenotypically-verified coreceptor usage was available demonstrated that the “Gly-Arg-Gly” crown motif is significantly more frequent in CXCR4-using C-HIV Envs (34.7%; n = 69) compared with R5 C-HIV Envs (0%; n = 428) (p<0.0001, Fisher’s exact test), as is a proximal “X-Gly” insertion at the same position as the “Ile-Gly” insertion shown in the X4 viruses of subject 1109 (33% of CXCR4-using Envs, n = 69; 0% of R5 Envs, n = 428) (p<0.0001, Fisher’s exact test). In addition, neither of these alterations occurred in any of the “final” R5 Envs from our panel. The “Gly-Arg-Gly” crown alteration also introduces an additional basic amino acid to the V3 loop, increasing the net charge of the V3 region. These V3 alterations are therefore likely to be particularly important for C-HIV coreceptor switching. Moreover, because previous studies have suggested that the gp120 V1/V2 loops might also be important for C-HIV coreceptor switching [63], we further hypothesized that the unique V1 loop sequence of the X4 variants in subject 1109 may influence coreceptor switching in this subject in concert with V3 loop alterations. These hypotheses were tested in the following mutagenesis studies.

### Identification of the Molecular Determinants of C-HIV Coreceptor Switching in Subject 1109

To better understand the molecular mechanisms contributing to C-HIV coreceptor switching, we next produced a panel of 15 Env mutants using the X4 1109-F-30 Env as template, introducing various V3 loop crown/stem alterations that are present in the R5 1109-E-10 Env, in the presence or absence of the V1 loop of the R5 1109-E-10 Env. The sequence alterations present in the Env mutants, which we term hereafter as M1 through M15 are shown in Figure 3B, and are described in detail in the Materials and Methods. For simplicity and to guide the interpretation of the subsequent results, their descriptions are summarized in Figure 3C.

**Figure 4:**
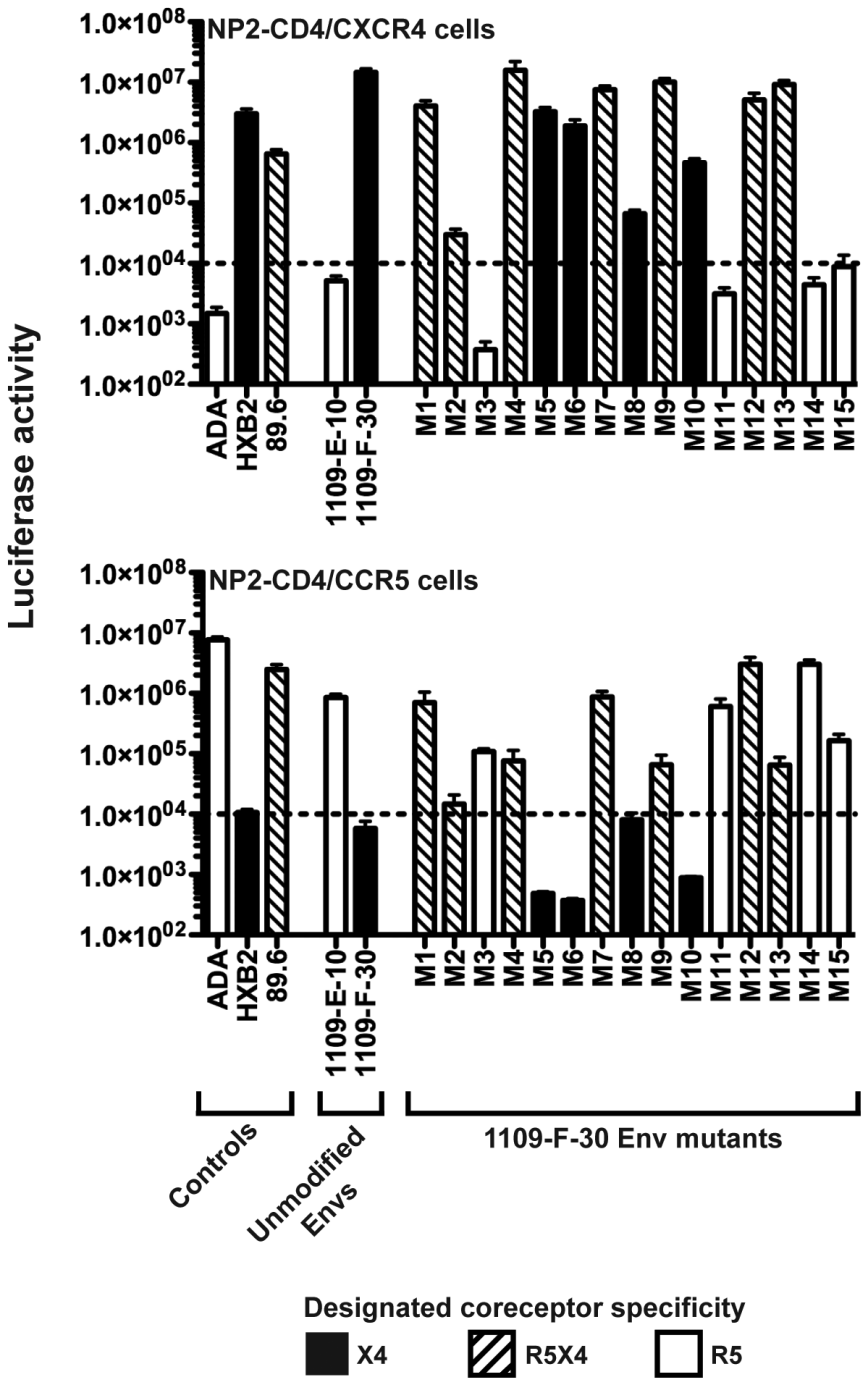
V3 loop alterations important for CXCR4 usage by C-HIV harbored by subject 1109. Luciferase reporter viruses pseudotyped with Env mutants (M1 through M15), unmodified 1109-E-10 and 1109-F-30 Envs, or control Envs (ADA, HXB2, 89.6) were used to infect NP2-CD4/CXCR4 and NP2-CD4/CCR5 cells, and the levels of HIV-1 entry were determined as described in Materials and Methods. Open bars indicate Envs with an R5 phenotype, black bars indicate Envs with an X4 phenotype, and hatched bars indicate Envs with an R5X4 phenotype. The dotted lines indicate the limit of detection of coreceptor activity. The results shown are a compilation of 3 independent experiments, each performed in triplicate, and each with an independent preparation of Env-pseudotyped virus. The data shown are means, and the error bars represent standard errors of the means.

We next produced luciferase reporter viruses pseudotyped with each of the Env mutants and compared their ability to enter NP2-CD4 cells expressing either CCR5 or CXCR4, relative to the unmodified 1109-E-10 (R5) and 1109-F-30 (X4) Envs (Fig. 4). Controls included ADA (R5), HXB2 (X4) and 89.6 (R5X4) Envs which as expected, efficiently entered NP2-CD4/CCR5 cells, NP2-CD4/CXCR4 cells, or both cell lines, respectively. The M5 and M6 mutants showed no affect on the X4 phenotype of 1109-F-30, suggesting that neither Asp331 nor the V1 loop of 1109-F-30 has a direct influence on the development of CXCR4-usage by this Env. Conversely, the M14 and M15 mutants completely abolished CXCR4 usage and conferred an R5 phenotype to 1109-F-30 Env; together with the lack of direct influence on coreceptor usage shown for the V1 loop, these results suggest that the principal determinants of coreceptor switching in this subject likely map to the V3 loop. The M1, M2 and M4 mutants restored CCR5 usage and conferred an R5X4 phenotype to 1109-F-30, suggesting that individually, acquisition of either Arg318, the Ile314-Gly315 insertion or Arg329 may confer an X4 phenotype from possible “transitional” R5X4 intermediates. The M3 and M11 mutants completely abolished CXCR4 usage and conferred an R5 phenotype to 1109-F-30; this suggests that acquisition of both the Ile314-Gly315 insertion and Arg318 is likely to be important for the transition of R5 to X4 phenotype in this subject, and further suggests that Arg329, while having an influence on coreceptor usage alterations in isolation, is not necessary for this transition.

To determine whether the Ile314-Gly315 insertion and Arg318 were sufficient to confer the X4 phenotype in subject 1109, we next produced an additional panel of 3 Env mutants using the R5 1109-E-10 Env as template, introducing these alterations either alone or in combination. The sequence alterations present in these Env mutants, which we term M16 through M18, are described in the materials and methods. These mutations, either alone or in combination, rendered 1109-E-10 Env completely non-functional for HIV-1 entry into NP2-CD4 cells expressing either CCR5 or CXCR4 (data not shown). These results suggest that the Ile314-Gly315 insertion and Arg318 of the X4 1109-F-30 Env are necessary but not sufficient for CXCR4 usage, and that the presence of additional “scaffold” mutations in 1109-F-30 Env, which may include changes in the V1 loop and/or other V3 loop changes (Fig. S2), is likely be required for Ile314-Gly315 and Arg318 to exert an influence on CXCR4 usage.

**Figure 5:**
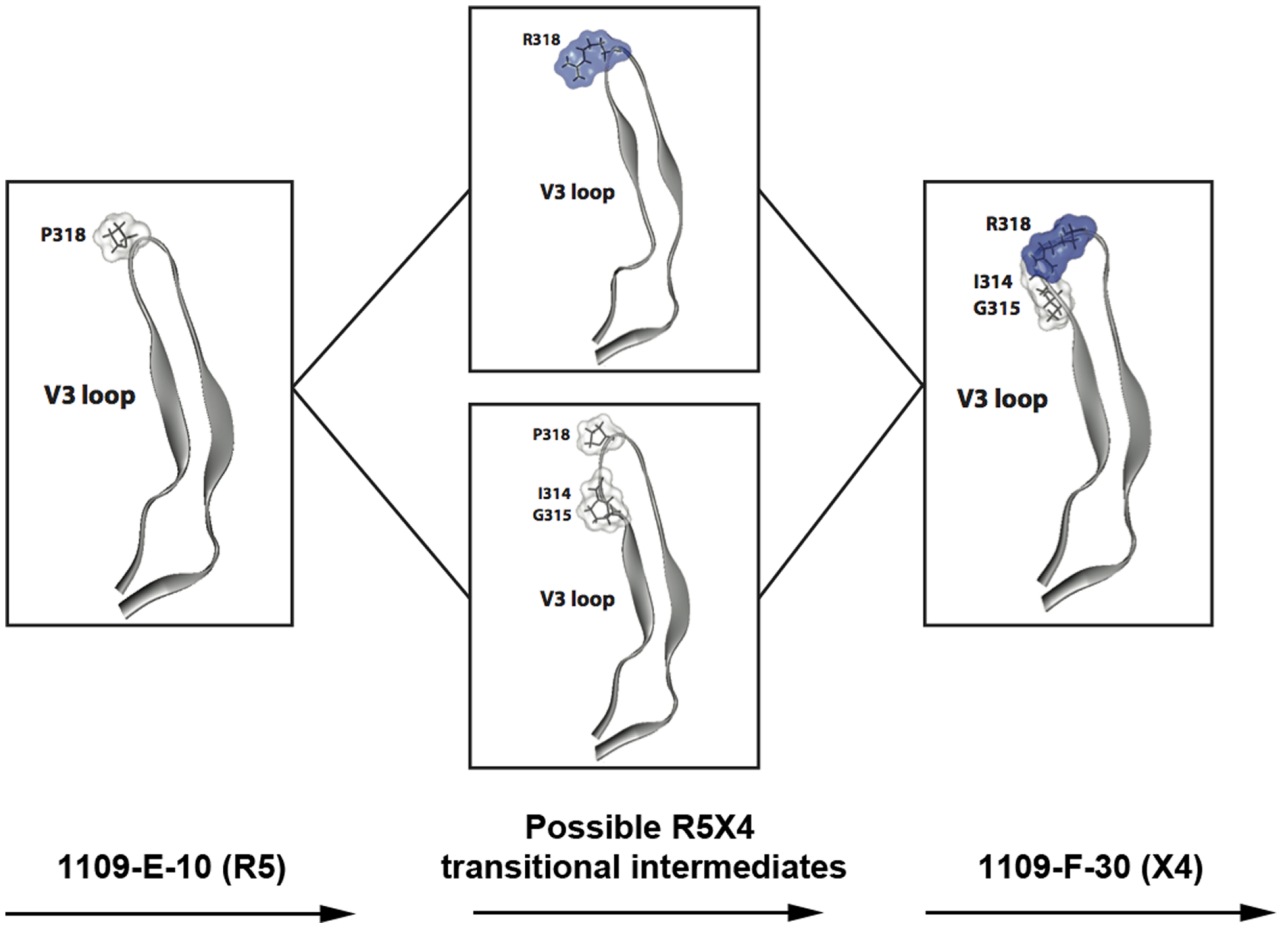
A model for the transition from R5 to X4 C-HIV phenotype in subject 1109. Close up views of the V3 loop structure of the R5 1109-E-10 gp120 (left), possible R5X4 intermediates (center, top and bottom) and the X4 1109-F-30 gp120 (right). Amino acid residues that contribute to the acquisition of CXCR4-usage in patient 1109, as determined by the Env mutagenesis studies, are represented as stick models and their molecular surface is colored according to polarity (white, non-polar; blue, positively charged). In this model, Envs that acquire either Arg318 (center, top) or the Ile314-Gly315 insertion (center, bottom) in the context of additional gp120 “scaffold” mutations are possible “transitional” R5X4 intermediates, with X4-phenotype conferred by the acquisition of both changes (right).

In summary these results support a model, illustrated in Figure 5, whereby in subject 1109, the development of CXCR4 usage occurs in the context of gp120 “scaffold” mutations and principally involves first, the acquisition of the Ile314-Gly315 insertion or Arg318, either of which on their own may confer possible “transitional” R5X4 phenotypes, and then the maintenance of both of these alterations to abolish CCR5 usage altogether and to confer the X4 phenotype.

## Discussion

By developing and characterizing the functional properties of a large longitudinal panel of Envs derived from clinically well-characterized ART-naïve subjects experiencing progression from chronic to advanced stages of C-HIV infection, our results highlight several important facets of C-HIV pathogenesis that reflect the natural history of progressive C-HIV infection. We show that the emergence of CXCR4-using strains at late stage, C-HIV infection was exceedingly rare in this untreated cohort, occurring in only one of the 21 subjects studied. However, further studies in larger cohorts of late stage, untreated subjects with C-HIV infection are required to confirm the results of our study. Moreover, although 6 independent X4 Envs were isolated from the late stage plasma sample of subject 1109, we cannot rule out the possibility that R5 and/or R5X4 variants were present as well, either as a minor subpopulation and/or by being selected against by the PCR. Nonetheless, when CXCR4-using variants emerged, the determinants of coreceptor switching were mapped principally to discrete alterations in the gp120 V3 loop region. In addition, we show that the significant decline in CD4+ T-cell counts, which fell to below or near 200 cells/µl defining immunodeficiency in nearly all subjects, was the result of infection by R5 C-HIV strains that persisted exclusively in 19/21 subjects. These results confirm that the pathogenic mechanisms of C-HIV infection that lead to immunodeficiency in the absence of antiretroviral intervention are indeed caused predominantly by R5 C-HIV strains, at least in the cohort studied. Importantly, our longitudinal Env panel can now enable future studies of Env determinants that contribute to C-HIV pathogenicity in the majority of subjects who do not experience a coreceptor switch.

Although switching coreceptor specificity to CXCR4-using variants was rare in late stage untreated C-HIV infection, deciphering the mechanisms involved in such coreceptor switching is important for understanding the complexity of virus-cell interactions in C-HIV pathogenesis. Our mutagenesis studies showed that the accumulation of two discrete amino acid alterations in the gp120 V3 loop, namely the Pro318Arg mutation at the V3 loop crown and the proximal Ile314-Gly315 insertion, were necessary for the transition of R5 to X4 phenotype by C-HIV harbored by subject 1109. However, further “gain-of-function” mutagenesis studies, whereby the Ile314-Gly315 insertion and/or Arg318 were introduced into the R5 1109-E-10 Env, showed that these changes, either by themselves or in combination, completely abrogated viral infectivity. These results suggest that the effects of Ile314-Gly315 and Arg318 on CXCR4 usage are context dependent, and most likely depend on the presence of additional “scaffold” mutations in the V1 and V3 loop regions. This interpretation is consistent with the results of recent studies which showed that a high level of genetic divergence in the gp120 V1/V2 region is required for C-HIV coreceptor switching, in addition to specific V3 loop alterations [63].

Database analysis of C-HIV Envs with phenotypically verified coreceptor usage, which included the analysis of C-HIV Envs sampled from divergent geographical regions and which included all of the independent CXCR4-using C-HIV Envs available in the Los Alamos database, showed that each of the V3 loop alterations that we showed were necessary for coreceptor switching in 1109-F-30 Env were present at relatively high frequency in CXCR4-using C-HIV Envs (Pro318Arg, 34.7%; X314-Gly315, 33%, n = 69), but that neither of these alterations were present in any of the R5 C-HIV Envs analyzed (n = 428). These results, which are supported by a recent cross-sectional analysis of CXCR4 using C-HIV Env sequences [64], provide compelling evidence that the Pro318Arg mutation and the X314-Gly315 insertion observed in 1109-F-30 Env may also be significant determinants of CXCR4-usage in other C-HIV strains. Further studies are required to determine if this is the case. However, in support of this possibility, using Env mutagenesis one previous study showed that acquisition of a X314-Gly315 insertion (which in this case was either a Met314-Gly315 or a Leu314-Gly315 insertion) could confer an R5X4 phenotype to C-HIV harbored by a pediatric subject, who was also ART-naïve when R5X4 variants developed [65]. These Env characteristics associated with CXCR4 usage, coupled with the increased net charge of the V3 loop due to the Pro318Arg mutation, could potentially be used to reliably predict CXCR4 usage of C-HIV Envs, and alleviate the need for functional phenotyping [58].

Not only do our mutagenesis studies shed light on the Env determinants contributing to the X4 C-HIV phenotype in subject 1109, from the combinations of mutations tested we were able to predict the determinants of likely “transitional” R5X4 intermediates. These intermediates were not detected in our longitudinal analysis of subject 1109, but most likely emerged after sampling of the “intermediate” plasma and were sequestered prior to sampling of the “final” plasma. Our mutagenesis study suggests that although acquisition of both the Pro318Arg mutation and the Ile314-Gly315 insertion (likely in the context of other gp120 “scaffold” mutations) were necessary for the X4 C-HIV phenotype, acquisition of either of these changes on their own could confer possible R5X4 intermediates (Fig.5). From our data we cannot determine the order in which these alterations may have appeared. However, because the M2 Env mutant dramatically reduced overall virus infectivity (Fig. 4), suggesting near-lethality conferred by the Pro318Arg alteration on its own, it would be reasonable to conclude that the Ile314-Gly315 insertion occurred before acquisition of Pro318Arg, in order for the virus to maintain high levels of infectivity during the transition from R5 to X4 phenotype. On the other hand, it is difficult to imagine the acquisition of such a dramatic structural alteration to the V3 loop as the primary instigator of coreceptor switching. We therefore cannot exclude the possibility that the Pro318Arg alteration occurred first, and that the more dramatic Ile314-Gly315 alteration occurred subsequently in an effort to rescue infectivity and confer the X4 phenotype. Further studies are required to more precisely determine the order of the acquisition of V3 loop mutations during the transition from R5 to X4 C-HIV phenotype.

One potentially important consideration for our study was that all the subjects had schistosomiasis at study enrolment, which may have influenced the patterns of coreceptor usage that we observed. Indeed, macaque studies have shown that helminthic parasitic infections can exacerbate infection with SIV due to alterations in the cytokine milieu and increased frequency of Th2 CD4+ T-cells (reviewed in [66]). Even after removal of these stimuli by effective treatment of the parasites, as was done with our study subjects at enrolment, their effects on HIV-1 replication may be long lasting. The Th2-type immune responses associated with schistosomiasis include elevated IL-4 and peripheral blood eosinophilia [66]. IL-4 may potentially down regulate CXCR4 expression [67]. Although it is unclear whether this would be sufficient to skew the emergence of CXCR4-using C-HIV variants in our cohort, we cannot rule out the possibility that immune responses associated with schistosomiasis had some influence on the evolution of the Env phenotypes in our study. On the other hand, there is strong overlap between regions of the world that are endemic for both helminthic and C-HIV infections [66], particularly in southern Africa which bears the brunt of the global HIV-1 pandemic, so it is reasonable to assert that the pathogenic viral processes illustrated by our cohort may indeed reflect the real life situation for the majority of individuals with C-HIV infection.

In conclusion, our results, that were generated using a unique longitudinal cohort of untreated subjects experiencing progressive C-HIV infection, provide new mechanistic insights into C-HIV pathogenesis. In addition, the extensive panel of Envs generated provides a valuable resource that builds capacity for new research into vaccines, novel inhibitors and microbicides with activity against C-HIV. To this end, it is our hope that the detailed supplementary section may be used as a catalogue for investigators to select and request specific reagents in order to expedite these critical areas of research.

## Supporting Information

Figure S1 **Intra-subject phylogenetic relationships of Env sequences.** Phylogenetic analysis of Env sequence sets for the individual subjects was conducted as described in the Materials and Methods. Sequence comparisons are made to reference HIV-1 subtype C Env sequences, and also to the reference HIV-1 subtype B Env sequence HXB2. Red circles represent Envs cloned from plasma taken at study enrolment; Green diamonds represent Envs cloned from the “Intermediate” plasma sample; Blue squares represent Envs cloned from the “Final” plasma sample. Refer to the main text for definitions of- and time frames associated with the Enrolment, Intermediate and Final plasma samples.
(TIF)

Figure S2 **Multiple sequence alignment of gp120 from “enrolment”, “intermediate” and “final” Envs obtained from subject 1109.** Multiple sequence alignment of Envs from subject 1109. Sequences are aligned to the 1109-E-10 clone. Dots indicate residues identical to 1109-E-10, and dashes indicate gaps. Boxed regions show amino acid changes in “final” (F) clones that could potentially be important for CXCR4 usage by these Envs.
(TIF)

Table S1 **Coreceptor usage and V3 loop sequence of Env clones.** The level of virus entry, based on consensus results from the two different cell systems, was scored as – (<5 fold above background),+(5–50 fold above background),++(50–300 fold above background), or+++(>300 fold above background). When CXCR4 usage was detected, infection in the CXCR4-expressing cell lines was repeated in the presence of 1 µM of the CXCR4 inhibitor AMD3100 to confirm specificity. E, I and F refer to Envs cloned from plasma obtained at study enrolment, approximately 1 year later (intermediate), and approximately 3 years after enrolment (Final), respectively. n/a; not applicable.
(TIF)
