# Supplementary figures and images for: Longitudinal Analysis of CCR5 and CXCR4 Usage in a Cohort of Antiretroviral Therapy-Naïve Subjects with Progressive HIV-1 Subtype C Infection
Source: PLoS One. 2013 Jun 18;8(6):e65950. doi: 10.1371/journal.pone.0065950 (PMC3688867; doi:10.1371/journal.pone.0065950)

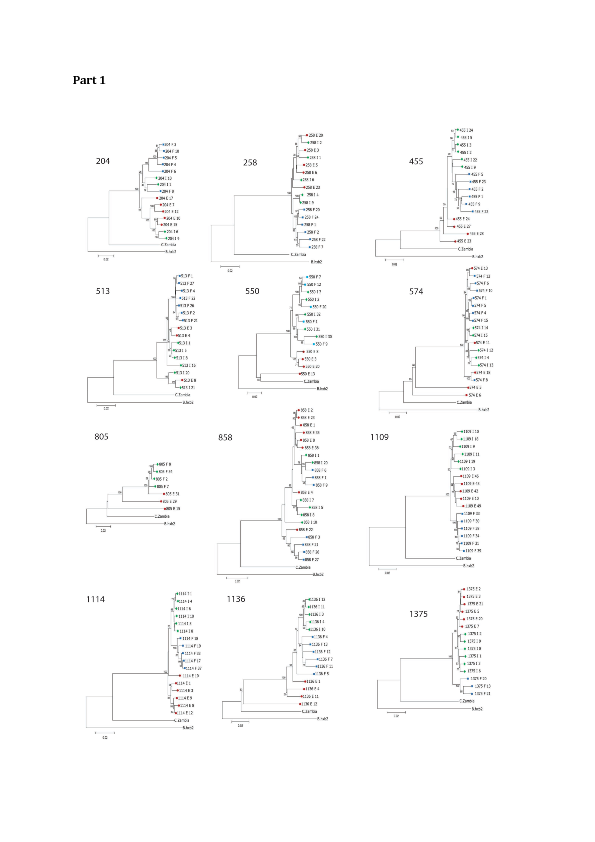

Supplement: Figure S1 — Intra-subject phylogenetic relationships of Env sequences. Phylogenetic analysis of Env sequence sets for the individual subjects was conducted as described in the Materials and Methods. Sequence comparisons are made to reference HIV-1 subtype C Env sequences, and also to the reference HIV-1 subtype B Env sequence HXB2. Red circles represent Envs cloned from plasma taken at study enrolment; Green diamonds represent Envs cloned from the “Intermediate” plasma sample; Blue squares represent Envs cloned from the “Final” plasma sample. Refer to the main text for definitions of- and time frames associated with the Enrolment, Intermediate and Final plasma samples. (TIF) [file pone.0065950.s001.tif]

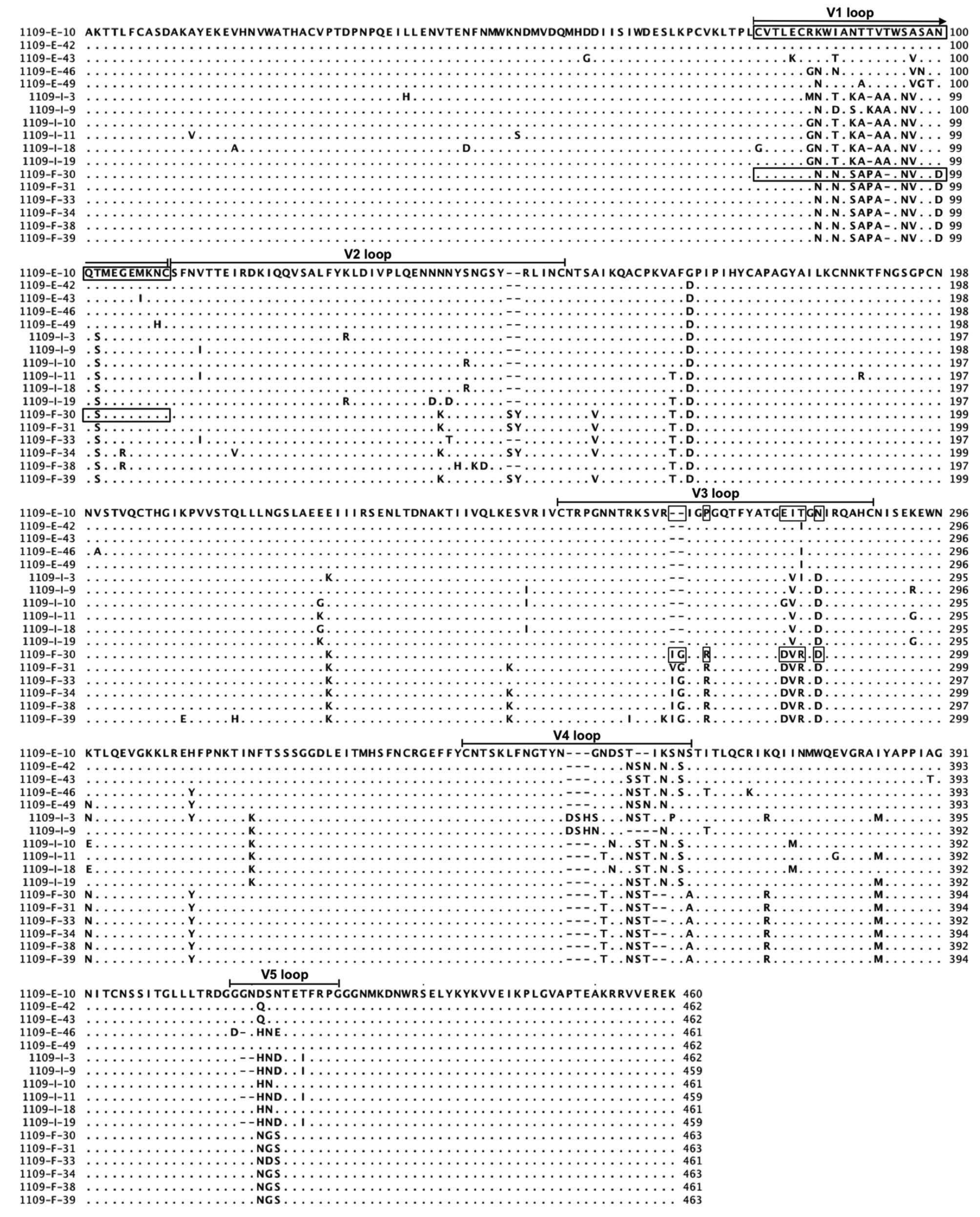

Supplement: Figure S2 — Multiple sequence alignment of gp120 from “enrolment”, “intermediate” and “final” Envs obtained from subject 1109. Multiple sequence alignment of Envs from subject 1109. Sequences are aligned to the 1109-E-10 clone. Dots indicate residues identical to 1109-E-10, and dashes indicate gaps. Boxed regions show amino acid changes in “final” (F) clones that could potentially be important for CXCR4 usage by these Envs. (TIF) [file pone.0065950.s002.tif]
